# Chikungunya in a kidney transplant recipient: a case report

**DOI:** 10.1590/2175-8239-JBN-2018-0196

**Published:** 2019-07-29

**Authors:** Renato Demarchi Foresto, Daniel Wagner de Castro Lima Santos, Maria Amélia Aguiar Hazin, Alejandro Túlio Zapata Leyton, Nayara Cordeiro Tenório, Laila Almeida Viana, Marina Pontello Cristelli, Hélio Tedesco Silva, José Osmar Medina Pestana

**Affiliations:** 1 Universidade Federal de São Paulo, Escola Paulista de Medicina São Paulo, SP, Brasil.; 2 Hospital do Rim, Divisão de Nefrologia, São Paulo, SP, Brasil.; 3 Hospital do Rim, Divisão de Doenças Infecciosas, São Paulo, SP, Brasil.

**Keywords:** Chikungunya virus, Kidney Transplantation, Arbovirus Infections, Immune Tolerance, Vírus Chikungunya, Transplante de Rim, Infecções por Arbovirus, Tolerância Imunológica

## Abstract

In 2004, a global spread of Chikungunya fever affected most tropical and subtropical regions of the world. In 2016, an outbreak occurred in Northeast Brazil with hundreds of cases documented. Solid organ transplant recipients have a modified immune response to infection and the clinical course is usually different from immunocompetent patients. The diagnosis can be challenging in this population. Most reports describe patients residing in endemic areas, although we must emphasize the importance of differential diagnosis in kidney transplanted travelers who visit endemic regions, such as Northeast Brazil. Here, we reported a case of a kidney transplant recipient that acquired Chikungunya fever after a trip to an endemic region at Northeast Brazil during the outbreak in 2016, with a good clinical evolution. We also present warning recommendations for travelers to endemic areas as additional measures to prevent disease outbreaks.

## RESUMO

Em 2004, um surto global de Chikungunya afetou a maioria das regiões tropicais e subtropicais do mundo. Em 2016, um surto ocorreu no Nordeste do Brasil com centenas de casos documentados. Receptores de transplantes de órgãos sólidos têm uma resposta imune modificada à infecção, e o curso clínico é geralmente diferente daquele em pacientes imunocompetentes. O diagnóstico pode ser desafiador nessa população. A maioria dos relatos descreve pacientes residentes em áreas endêmicas, embora devamos enfatizar a importância do diagnóstico diferencial em viajantes transplantados renais que visitam regiões endêmicas, como o Nordeste do Brasil. Aqui, nós relatamos o caso de um receptor de transplante renal que adquiriu febre Chikungunya após uma viagem a uma região endêmica no Nordeste do Brasil durante o surto de 2016, com uma boa evolução clínica. Também apresentamos recomendações de alerta para viajantes em áreas endêmicas, como medidas adicionais para prevenir surtos de doenças.

## INTRODUCTION

Chikungunya fever is a disease caused by the arbovirus Chikungunya (CHIKV), belonging to the Togaviridae Family and the *Alphavirus* genus. CHIKV is transmitted by the bite of the mosquito vectors *Aedes aegypti* or *Aedes albopictus* 1. The name “Chikungunya” comes from the Makonde language in Tanganyika (Tanzania) and means “bended person” or “the one who stoops”, describing the posture caused by the severe arthralgia present in patients with Chikungunya fever^2^. CHIKV is endemic in certain parts of West Africa, East Asia, and Latin America, and some outbreaks of Chikungunya disease have occurred in Brazil in the last years. Autochthonous cases in Brazil started in 2014, with a major outbreak in the Northeast region in mid-2016^3^.

After a short period of incubation of 3 to 7 days, the main symptoms begin abruptly with severe polyarthralgia, fever above 39°C, diffuse maculopapular rash, asthenia, myalgia, and headache, with self-limiting duration of around 7 to 10 days. Polyarthralgia and myalgia can last several weeks to even months, leading to chronic weakness. The most common serum abnormalities are lymphopenia, thrombocytopenia, elevated CPK, mild elevated liver enzymes, and mild to moderate acute kidney injury^4^.

Although Chikungunya is not a disease of high lethality, it has an endemic behavior with a high morbidity rate associated with persistent arthralgia, resulting in low quality of life. Severe manifestations include septic shock, myocarditis, meningoencephalitis, hepatic failure, respiratory distress, renal failure, and cryoglobulinemia. Although these complications and death are rare, they occur more often in patients over 65 years of age^5^^,^ 6. Chronic joint symptoms may occur in 25-35% of non-transplanted patients^1^. The clinical manifestations have a specific evolution in patients who received immunosuppressive drugs, as solid organ transplant recipients, with a few cases described in literature^7^^,^ 8. Supportive care is the standard treatment to Chikungunya fever and specific antivirals are not available.

Chikungunya infection in kidney transplant recipients presents atypical clinical evolution and prognosis compared to infection in immunocompetent patients. Despite the occurrence of an outbreak of the disease in 2016, there are few reported cases in transplant recipients, notably in transplanted travelers, probably a consequence of underdiagnosis and underreporting. This case report corroborates the clinical findings of the Chikungunya infection described in kidney transplant patients, and alerts on the recommendations for those patients traveling to endemic areas.

## CASE REPORT

A 42-year-old woman, submitted to living donor kidney transplantation 7 years before, was admitted to the hospital due to fever (up to 40°C) since the day before admission, associated with severe pain in knees and right shoulder, and one episode of diarrhea 5 days before. She referred a trip to the countryside of Paraiba, at Northeast of Brazil, 20 days before admission, where there was a local outbreak of Chikungunya. Physical examination revealed no signs of arthritis, cutaneous rash and respiratory distress. Neurological exam was normal.

Results of exams collected at admission revealed: creatinine 1.64 mg/dL (baseline 1.00 mg/dL); hemoglobin 12.1 g/dL; hematocrit 36.7%; leukocytes 7,000/µL (differential counts of neutrophils 6,230/µL; lymphocytes 280/µL; basophils 0/µL; eosinophils 0/µL; monocytes 490/µL), platelets 125,000/µL; C-reactive protein 8.08 mg/dL; creatine phosphokinase 143 U/L; alanine aminotransferase (ALT) 81 U/L; aspartate aminotransferase (AST) 55 U/L; NS1 rapid test for dengue was negative; serology for Chikungunya IgM was positive, and qualitative reverse transcription polymerase chain reaction (RT-PCR) was detectable. The patient was taking a combination of immunosuppressive drugs with prednisone 5 mg once a day, tacrolimus 5 mg bid, and mycophenolate 720 mg bid.

During hospitalization, after the diagnosis of Chikungunya fever, the dose of mycophenolate was reduced to 360 mg bid and the sulfamethoxazole-trimethoprim for pneumocystosis prophylaxis was stopped due to lymphopenia. The patient received venous and oral hydration, with recovery of graft function. The medical team opted not to increase corticoid dose. The patient had a good clinical evolution, with return to baseline graft function and clinical improvement. After a 2-year follow-up, the patient remained with good renal function (creatinine of 0.92 mg/dL), but with complaints of body cramps until 1 year after the infectious episode. The patient had no bone deformation or chronic arthritis.

## DISCUSSION

CHIKV and other arboviruses such as Dengue, Zika and Yellow fever (YF) can infect people living in or traveling to endemic areas in Latin America, Africa, and East Asia. There are few data about arboviruses in solid organ transplant recipients, but the real incidence of these infectious diseases is probably greater than we think^9^^,^ 10. There is no reported case of YF transmitted by blood transfusion or organ transplantation, probably due to the vaccine against YF. Transmission of the other arboviruses by these routes is anecdotic^11^.

Since the global spreading of CHIKV in 2004, many countries situated in tropic and subtropic regions have endemic CHIKV circulation. In Brazil, *Aedes aegypti* is disseminated in all regions and widely dispersed in urban areas, where cases of Chikungunya fever have already been documented^12^.

Mosquitoes become infected when they feed on a person infected by the virus, which reaches its salivary glands and is inoculated in the next person bitten by the insect. The virus life cycle involves human and other primates that live in the jungle, although urban transmission can occur due to mosquito habits. The vectors *Aedes aegypti* and *Aedes albopictus* also transmit Zika virus and Dengue virus, so they have the same endemic areas of transmission, and coinfections have been documented^10^^,^ 13. CHIKV may also be transmitted via maternal-fetal blood infection and rarely via solid organ transplantation, only if viremia is above 10^4^-10^9^ RNA copies/mL and the recipient has a lack of specific IgM and IgG response^14^.

CHIKV infection should be suspected in patients with acute onset of fever and polyarthralgia, and documented epidemiologic exposure. The laboratory diagnosis opportunity depends on the period of the infection. During the viremic period, usually up to one week from the onset of the symptoms, the diagnosis of the acute infection is based on direct methods, such as RT-PCR. Reported sensitivity and specificity of the CHIKV-IgM ELISA assay (Euroimmun^®^) are 98 and 97.5%, respectively^15^.

Chikungunya infection can be a cause of acute kidney injury (AKI) in severe cases, mainly secondary to pre-renal lesion (20%), but there are few reports of AKI due to rhabdomyolysis and acute interstitial nephritis^16^^,^ 17. Our patient had a mild transient KDIGO 1 AKI attributed to pre-renal lesion, with complete response after intravenous fluids. Rhabdomyolysis was discarded.

The patient described above was a kidney transplant recipient infected by a mosquito bite. Autochthonous cases in Brazil started in 2014, with a major outbreak in the Northeast region in mid-2016, probably when the described patient acquired the disease. Two articles published by Pierotti et al. and Girão et al. describe 4 and 9 cases, respectively, of Chikungunya fever in kidney transplant recipients, all of them with good evolution, without severe symptoms, such as in the case presented above^7^^,^ 8.

The literature has conflicting data about the clinical course of persistent polyarthralgia in patients using immunosuppressive drugs. These symptoms are generally incapacitating and have a negative impact in the quality of life of this population. In a cohort of 180 patients in the general population, 60% had severe persistent arthralgia or joint swelling at 3 years after the acute infection^18^. Economopoulou et al. in 2009 described a cohort of 610 atypical cases with only 3 of them having kidney transplants, and Kee et al. in 2010 described 2 cases of Chikungunya fever with atypical course and bacterial coinfections^16^^,^ 19.

Previously published data showed that patients who underwent immunosuppression, like kidney transplant recipients, were more likely to have bacterial coinfections and poor clinical results^16^^,^ 20 ^,^ 21. However, some recent retrospective cohorts demonstrate that solid organ transplant recipients have a good clinical course of Chikungunya fever, with less incidence of severe manifestations and persistent arthralgia, possibly due to the use of corticosteroids for immunosuppression, as suggested by other authors^7^^,^ 8.

The two main symptoms of the disease are fever and arthralgia. Both are associated to the synthesis of cytokines such as interleukin-1b, IL-6, and tumor necrosis factor-alpha, which are known pyretics and cause inflammation or destruction of cartilage, synovial, and bone tissues. It has also been shown that interferon (IFN) produced by CHIKV-infected fibroblasts induce high expression of prostaglandins in these patients. Immunosuppressive drugs such as calcineurin inhibitors decreases the expression of genes encoding proinflammatory cytokines (interferon, TNF-alpha). Corticosteroids inhibit lymphocyte proliferation and cytokine synthesis, and mycophenolate exhibit a marked antiproliferative effect in all mononuclear cells, inhibiting the production of IL-1 alpha, IL-1 beta, IL-2, IL-3, IL-4, IL-5, IL-6, IL-10, interferon gamma (IFN-gamma), and tumor necrosis factor alpha (TNF-alpha). Thus, it is possible that anti-rejection drugs may cause a blockage of cytokines production that cause inflammatory responses in Chikungunya fever, decreasing the expression of symptoms and the severity of cases^22^^,^ 23.

Some guidelines recommend a short period of corticosteroids for treating inflammatory manifestations of CHIKV, like polyarthritis, during 1 to 2 months, with low-dose prednisone (5-10 mg)^24^^-^ 26. Rheumatologists recommend the use of hydroxychloroquine or meloxicam for persistent polyarthralgia instead of other nonsteroidal anti-inflammatory drugs^26^, but there is no evidence of benefit in solid organ transplant recipients. In addition, the use of NSAIDs is strongly contraindicated in kidney transplant recipients, due to the risk of acute graft dysfunction.

No antiviral has been approved for treatment of CHIKV fever, but the combination of doxycycline and ribavirin showed a potential effect on inhibiting entry and replication of CHIKV in culture of kidney epithelial cells extracted from monkeys, and also reduced viral infectivity *in vitro* and *in vivo* 27. Human vaccine against CHIKV is not yet available for clinical practice; notwithstanding, inactivated and attenuated vaccine candidates have been studied in phase I/II trials, and engineered vaccines have proven to be safe and immunogenic in mouse and nonhuman primate models^28^.

Further studies are needed to determine a suitable treatment for both the eradication of serum CHIKV circulation and the management of chronic symptoms. Meanwhile, environmental measures already implemented in campaigns to combat *Aedes aegypti* proliferation can help control and prevent CHIKV epidemics in tropical countries, such as Brazil. The Brazilian Ministry of Health recommends as prevention measures for travelers to endemic areas the use of repellents in exposed skin areas, keeping doors and windows closed if possible, wearing clothing that protects skin surface, such as pants and a long-sleeved shirt, socks, and closed shoes, avoiding environments with mosquitoes without the above protective measures, and looking for areas of mosquito breeding nearby and notifying the person responsible for their proper elimination. If a traveler develops the disease, he or she becomes a potential reservoirs of infection for other people and should maintain the above protective measures to avoid disease spreading. Most importantly, the person should seek medical advice immediately and avoid self-medication^29^.

Here, we alert clinicians and nephrologists about the paucity of symptoms of arbovirosis like Chikungunya fever and advise early suspicion and correct treatment. Furthermore, patients traveling to areas at risk should be instructed to maintain clinical surveillance and seek medical attention in the event of fever, arthralgia, rash, headache, and fatigue for up to 7 days after the trip.

## CONCLUSION

We presented a case of an immunosuppressed kidney transplant recipient with Chikungunya fever, with a non-specific clinical course, demonstrating that this disease should be part of the differential diagnosis of febrile syndrome and polyarthralgia in transplanted patients in an endemic country. We have also given recommendations for travelers to endemic areas as additional measures to prevent disease outbreaks.
